# A New Window onto the Pacemaker of the Heart, the Sinus Node, Provided by Quantitative Proteomics and Single-Nucleus Transcriptomics

**Published:** 2020

**Authors:** Mark Boyett, Alicia Lundby

**Affiliations:** 1 Department of Biomedical Sciences, Faculty of Health and Medical Sciences, University of Copenhagen, København, 2200, Denmark; 2 The Novo Nordisk Foundation Center for Protein Research, Faculty of Health and Medical Sciences, University of Copenhagen, København, 2200, Denmark

Hypothesis-driven research has dominated biomedical science for at least the past century [1]. There are many papers and grant applications that will have been rejected because they are not hypothesis-driven. For example, Haufe [2] reports that the NIH guidelines for RO1 grants states that “A strong grant application is driven by a strong, solid hypothesis with clear research objectives”. More than 50 years ago, the Austrian-born British philosopher Karl Popper formalised this [3]. He believed that good science involves rigorously testing a hypothesis to determine its validity; when the hypothesis fails, a new hypothesis is formulated and after this the process is repeated [3]. The advantage of hypothesis-driven research is that it channels the investigator’s efforts, and it provides a framework for progressing our knowledge in a methodical stepwise manner. Undoubtedly, hypothesis-driven research is important and will continue to be so. However, hypothesis-driven research also blinkers the investigator to the complexity of nature and the temptation is for the investigator to attribute a phenomenon to one factor, which is rarely the case – nature is too complex. The alternative to hypothesis-driven research is hypothesis-generating or exploratory research and the advantage of this is that it does not constrain the view of the investigator. The amassing of enormous data sets in genomics, transcriptomics and proteomics as well as automated data-mining techniques have facilitated this alternative approach, resulting in ‘data-driven discovery’ [1].

The article by Linscheid et al. [4] heralds the arrival of data-driven discovery to the study of the sinus node and provides an example of this alternative approach. The sinus node is the pacemaker of the heart. It is responsible for the initation of the cardiac action potential and therefore the heart beat [5]. It is located in rear wall of the right atrium where it meets the superior vena cava and it is made up specialised myocytes with a distinct embryological origin to the working muscle of the heart [5]. It is not an endocrine tissue (unlike the neighbouring atrial muscle which secretes atrial natriuretic peptide), but it is highly regulated by neurohumoral signalling. What has fascinated investigators since the discovery of the sinus node over 100 years ago is the mechanism underlying pacemaking. The rhythmic pulse of blood flow through the sinus node artery has even been suggested to be responsible [6], but traditionally research has focused on a handful of ionic currents and underlying ion channels responsible for pacemaking. This narrow view has now changed. Mass spectrometry based proteomics technologies have advanced our understanding of cellular signalling [7,8] as well as the molecular build-up of cells [9]. The strength of proteomics lies in its unbiased approach to investigate the architecture of protein and signaling networks of a given biological system. We have optimised methods to apply the technology to study cardiac tissue samples; which covers specific strategies for tissue homogenisation, protein extraction and enzymatic digestion into peptides [10,11]. We pre-fractionate peptides at high pH utilizing high-capacity offline HpH reversed-phase liquid chromatography, which enables deep proteome measurements [12]. Cardiac samples are measured on a Quadrupole Orbitrap mass spectrometer to achieve deep, and quantitative, cardiac proteomes. Using such a high-resolution mass spectrometry based proteomics approach, we measured and quantified the expression of 7,248 proteins in the sinus node and atrial muscle. Studying thousands of proteins in a tiny cardiac region such as the sinus node presents a holistic picture of the molecular composition of this particular cardiac region, and offers the possibility to identify which parts of the molecular landscape is unique to the sinus node. We observed significant differences in the expression of 575 proteins between the sinus node and atrial tissues; a much more complex finding than could have been evaluated by a hypothesis-driven approach.

Pacemaking is said to be the result of a membrane clock and a Ca^2+^ clock and the relative importance of the two is controversial [13]. The quantitative proteomics data reveal significant differences in the expression of the ion channels responsible for the membrane clock between the pacemaking sinus node and non-pacemaking atrial muscle, but not in Ca^2+^ clock proteins, suggesting that the membrane clock underpins pacemaking; pacemaking ion channels are more highly expressed in the sinus node. The hyperpolarization-activated cyclic nucleotide gated ion channel, HCN_4_, is arguably the most important component of the membrane clock and quantitative proteomics shows that it is more than 500 times as abundant in the sinus node as in the atrial tissue; in fact it is the most differentially expressed protein (of the 7,248 proteins detected) in favour of the sinus node. We used a combination of Markov chain modelling of ion channels and the quantitative proteomics to estimate that there are 2,227 HCN_1_ channels and 6,255 HCN_4_ channels (important components of the membrane clock) and 16,079 sarcoplasmic reticulum RyR2 Ca^2+^ release channels and 6,630,737 sarcoplasmic reticulum Serca2 Ca^2+^ pumps (important components of the Ca^2+^ clock) per sinus node myocyte. These findings exemplifies how the large scale data generated with proteomics technologies can be translated into specific findings of individual proteins, just as we know it from traditional hypothesis-driven research.

The functions of the 575 proteins that are differentially expressed between the sinus node and atrial muscle go beyond pacemaking and ion channels. Proteins with higher expression in the sinus node are enriched for functions related to actin cytoskeleton, contractile fibres, chromatin, neurofilament, carbohydrate metabolism, lipid metabolism, collagen and connective tissue [4]. These functional groups represent the unique sinus node architecture at the molecular level. They show for example that in the sinus node:

- gap junctions are more poorly expressed – this is presumably to protect the pacemaker tissue of the sinus node from the hyperpolarizing (and pacemaker-suppressing) action of the surrounding atrial muscle [14].
- some contractile proteins are more highly expressed – this includes the contractile proteins, Myh6 and Myl4, linked to sinus node dysfunction [15,16].
- proteins of the natriuretic peptide system are more poorly expressed – the natriuretic peptide system and the secretion of atrial natriuretic peptide is known to be a property of the atrial muscle and this shows that it does not extend to the sinus node.
- proteins involved in lipid storage are more highly expressed – lipids are the primary fuel source for myocytes and, therefore, this may be beneficial for pacemaking.
- there is a unique expression pattern of transcription factors – with a unique pattern of gene transcription in the sinus node, this is expected.
- there is more neuronal innervation – it has long been known that the sinus node is highly innervated [17].
- the extracellular matrix is characteristic of highly elastic tissue - this presumably affords protection to the sinus node, which is located in the thin rear wall of the right atrium and subject to regular distension as blood moves in and out of the chamber.

The sinus node is not made up of a single cell type. The pacemaker cells are the most studied cell type of the sinus node, but the node is made up of multiple cell types, all of which are likely to contribute to sinus node physiology and pathophysiology. In this study, as well as quantitative proteomics, we used single nucleus RNAseq to read the transcriptomes of sinus node cell types and we identified 12 different cell populations. Among the 12 cell populations of the node were three types of adipocytes and two types of fibroblasts. The study of Linscheid et al. [4] presents how omics technologies can be applied to open new windows onto sinus node discoveries and also the search for new treatments of sinus node node disease. For example, in ageing, heart failure, atrial fibrillation, pulmonary hypertension, thyroid disease and athletes, and at night-time, there is sinus node dysfunction [5,18] and this is likely to involve many processes such as ion channel disarray e.g. [19], downregulation of autonomic innervation and metabolism [20–23], activation of the CaMKII pathway [24], a changing pattern of protein phosphorylation, fibrosis, apoptosis, inflammation, transcription factors and microRNAs, all of which are measurable by the new omics technologies. Of course the advantages of the new omics technologies and data-driven discovery are not just restricted to the study of the sinus node, and there will be ample opportunities for their use in for instance the field of immunology.
